# Supplementary material for: Identification of photoperiod-induced specific miRNAs in the adrenal glands of Sunite sheep (Ovis aries)
Source: Front Vet Sci. 2022 Jul 22;9:888207. doi: 10.3389/fvets.2022.888207 (PMC9354845; doi:10.3389/fvets.2022.888207)
Supplement: Supplementary file 1 [file Data_Sheet_1.ZIP › Supplementary file/miRDeep2 file.zip!/miRDeep2 file/Novel_Structure/SP42A3/Novel_41.pdf]

Provisional ID : NC_019469.2_18836
Score total : 14.5
Score for star read(s) : 3.9
Score for read counts : 7.8
Score for mfe : 1.2
Score for randfold : 1.6
Score for cons. seed :
Total read count : 27
Mature read count : 15
Loop read count : 0
Star read count : 12

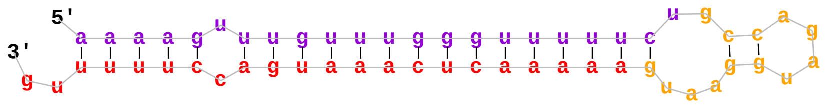

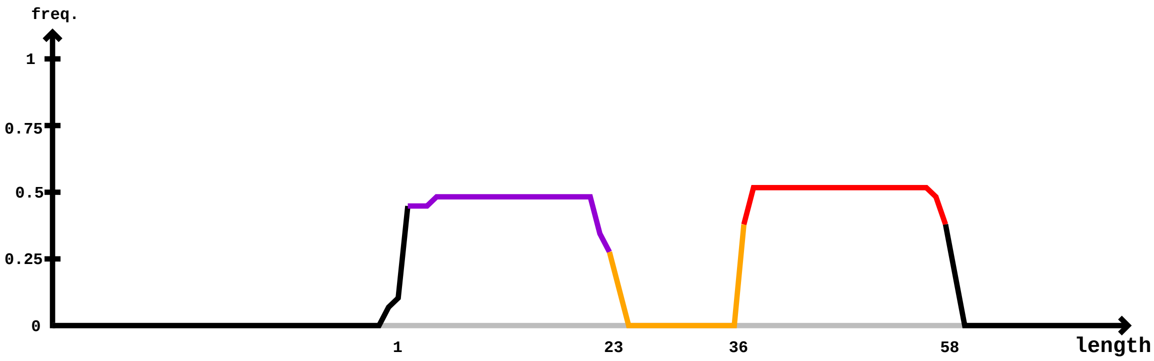

Star Mature

```
5'- caagucauuguaaaacugcuaguauuggcuuggccaaaaguuuguuuggguuuuucugccagauggaaugaaaaacucaaaugaccuuuuuggccaacuugguauuuuua  -3'   obs
    caagucauuguaaaacugcuaguauuggcuuggccaaaaguuuguuuggguuuuucugccagauggaaugaaaaacucaaaugaccuuuuuggccaacuugguauuuuua         exp
    (((((............((((....)))).((((((((((.(((((((((((((((..((....))...)))))))))))))))).).))))))))))))))).........   reads    mm     sample
    .................................cAaaaaguuuguuuggguuuuu.......................................................   2        1      seq
    ..................................Aaaaaguuuguuuggguuuuucu.....................................................   1        1      seq
    ...................................aaaaguuuguuuggguuuCu.......................................................   1        1      seq
    ...................................aaaGguuuguuuggguuuuu.......................................................   1        1      seq
    ...................................aaaUguuuguuuggguuuuuc......................................................   1        1      seq
    ...................................Gaaaguuuguuuggguuuuuc......................................................   1        1      seq
    ...................................aaaaguuuguuuggAuuuuucu.....................................................   1        1      seq
    ...................................aaaaguuGguuuggguuuuucu.....................................................   1        1      seq
    ...................................aaaaguuuguuuggguuuuucu.....................................................   3        0      seq
    ...................................Caaaguuuguuuggguuuuucu.....................................................   1        1      seq
    ......................................aguuuguuuggAuuuuucug....................................................   1        1      seq
    ......................................................................aaaaacucaaaugaccuuuu....................   1        0      seq
    ......................................................................aaaaacucaaaugaAcuuuuu...................   3        1      seq
    ......................................................................aaaaacucaaaugaAcuuuuug..................   6        1      seq
    ......................................................................aaaaacucaaaugaAcuuuuugg.................   1        1      seq
    .......................................................................aaaacucaaaugaAcuuuuug..................   4        1      seq
```
